# Spatiotemporal Characteristics, Determinants and Scenario Analysis of CO_2_ Emissions in China Using Provincial Panel Data

**DOI:** 10.1371/journal.pone.0138666

**Published:** 2015-09-23

**Authors:** Shaojian Wang, Chuanglin Fang, Guangdong Li

**Affiliations:** 1 School of Geography and Planning, Sun Yat-Sen University, Guangzhou, 510275, China; 2 Institute of Geographic Sciences and Natural Resources Research, Chinese Academy of Sciences, Beijing, 100101, China; 3 Key Laboratory of Regional Sustainable Development Modeling, Chinese Academy of Sciences, Beijing, 100101, China; Shandong University, CHINA

## Abstract

This paper empirically investigated the spatiotemporal variations, influencing factors and future emission trends of China’s CO_2_ emissions based on a provincial panel data set. A series of panel econometric models were used taking the period 1995–2011 into consideration. The results indicated that CO_2_ emissions in China increased over time, and were characterized by noticeable regional discrepancies; in addition, CO_2_ emissions also exhibited properties of spatial dependence and convergence. Factors such as population scale, economic level and urbanization level exerted a positive influence on CO_2_ emissions. Conversely, energy intensity was identified as having a negative influence on CO_2_ emissions. In addition, the significance of the relationship between CO_2_ emissions and the four variables varied across the provinces based on their scale of economic development. Scenario simulations further showed that the scenario of middle economic growth, middle population increase, low urbanization growth, and high technology improvement (here referred to as Scenario BTU), constitutes the best development model for China to realize the future sustainable development. Based on these empirical findings, we also provide a number of policy recommendations with respect to the future mitigation of CO_2_ emissions.

## Introduction

Global climate change—specifically, global warming—constitutes one of the most important issues to face human beings in the 21^st^ century [1]. That greenhouse gases, most notably carbon dioxide (CO_2_) emissions from the combustion of fossil fuel, are the main influencing factor of global warming which is now a matter of global consensus and, since CO_2_ emissions are closely related to socioeconomic development, climate change has gone from being an issue for pure scientific research to becoming an international political, economic and diplomatic hot topic [2]. Locating the key influencing factors of CO_2_ emissions, in order to effectively curb these emissions, is an increasingly important task, especially for China, the world’s largest developing country [3]. Studies show that China—as the world’s largest energy consumer, consuming nearly half of all coal produced in the year of 2008, and the world’s highest CO_2_ emitter, accounting for one-quarter of the world’s CO_2_ emissions in the year of 2011 –has been responsible for 80% of global increases in CO_2_ emissions since 2008 [4]. It is therefore vitally important that an optimal development model be identified which has the capacity to enable China to reduce CO_2_ emissions while maintaining economic growth. It is now generally recognised that energy consumption is the key impact factor of CO_2_ emissions; in order to realise CO_2_ emissions reduction targets, though, we also need to examine other important influencing factors in relation to CO_2_ emissions. Consequently, the following questions are critical to China’s sustainable development: How does the scale of the population, the economic level, energy intensity, and the level of urbanization affect CO_2_ emissions? Do CO_2_ emissions continue to growth rapidly? Modelling and forecasting China’s CO_2_ emissions would allow us to determine the optimal development model for future socioeconomic development.

Studies are increasingly being undertaken in order to examine the factors that affect CO_2_ emissions in a range of different countries and regions [5]. The existing literature addressing the influencing factors of CO_2_ emissions mainly falls into four categories, which can be differentiated in terms of different methods used in their studies. The first category of studies rely upon the stochastic impact by regression on population, affluence (GDP) and technology (IPAT) model, in its extended form—the STIRPAT model. The STIRPAT (IPAT) model is one of the most popular methods for exploring the impact factors of CO_2_ emissions, and it has been used in a large range of studies—for instance, in studies undertaken by York et al. [6] and Shi [7], who adopted the model in order to estimate the influence of population scale on CO_2_ emissions; in Xu and Chen [8], a study which analysed the impact of population and affluence on emissions in China; in Fan et al.’s [9] exploration of the influence of population, GDP and technology on the CO_2_ emissions of countries fallen into different groups based on income levels; and in Liu and Liu’s [10] study of the determinants of CO_2_ emissions in China. Similar studies have also been also undertaken by Lin et al. [11],Yan et al. [12], Shao et al. [13], Sun et al. [14], Li et al. [15], Wang et al. [16] and Li et al. [17]. More recently, using an improved STIRPAT model, Wang et al. [18] found that the urbanization, GDP and industry structure all exerted a positive impact on CO_2_ emissions in Beijing city, while tertiary industry proportion, energy intensity and R&D output negatively influenced CO_2_ emissions. These results are supported by a number of Chinese studies—for instance those undertaken in relation to Jilin province by Ma et al. [19], Guangdong province by Wang et al. [20] and in Tianjin city by Zhao and Guo [21].

The second category comprises of research that has used structural decomposition analysis (SDA) and index decomposition analysis (IDA), mainly the Logarithmic Mean Divisia Index (LMDI) model based on time series data. This group of studies includes Ang et al. [22], Hoekstra and Van [23], Wang et al. [24], Wu et al. [25], Ma and Stern [26], Elif et al. [27], Song [28], Zhang et al. [29], Brizga et al. [30], Gui et al. [31] and Xu et al. [32]. Various index decomposition methods, based on decomposition formulations and index numbers, have been employed in this particular body of literature. In these studies, CO_2_ emissions have generally been decomposed, using the Kaya Identity model, into population scale, economic scale, energy intensity, energy consumption structure, industrial structure and carbon intensity [33]. Most studies of this character have found energy intensity and the economic level to be the key influencing factors in relation to CO_2_ emissions, in comparison to which economic structure, industry structure and energy structure have been found to have a lesser impact.

The third category is constituted by studies that have relied on panel data analysis. Typically, this method has been used to examine the influencing factors of CO_2_ emissions based on panel data. It is recognized that panel data models have many metrics over conventional cross-sectional and time series data models [34]. Recently, using the method of panel data analysis, Dinda and Coondoo [35] and Narayan and Narayan [36] analysed the relationship between economic growth and CO_2_ emissions, and Wang et al. [37], Ozkan and Ozkan [38], Zhu et al. [39] and Ozcan [40] have also attempted comprehensive analyses of the impact factors of CO_2_ emissions, finding population and economic level to be the main factors affecting those emissions.

The fourth category of studies is made up of research using a range of other determinant analysis models. The representative analysis methods in this group include: the input–output model [9, 41], the Laspeyres method [42], the AWD method [43], the GFI method [44], the ARDL model [45–46], the LR-MEF (long-run marginal emissions factor) method [47] and the Decoupling index formulation [48]. These methods have provided new insights into the influence mechanisms in relation to CO_2_ emissions, and have broadened our methodological perspectives.

These existing studies have enriched our understanding of the main human impact factors in CO_2_ emissions. However, most of the literature has been inclined to analyse influence mechanisms at a cross-country level [34], at a single-city level or at a national level [18, 20], and only a handful of studies have addressed the cross-province level of China. In addition, despite the fact that it is recognized that panel data sets have many metrics over conventional cross-sectional or time series data sets [33–34, 49], most models used in simulating China's CO_2_ emissions have been based on country or city level time series or cross-sectional data [33], with only a few studies being based on panel data models [2, 33]. More importantly, existing studies have mainly focused on estimating the influencing factors of CO_2_ emissions, seldom proceeding to analyse the development models that might promote a country’s sustainable development.

Based on the above review, this study first calculated CO_2_ emissions for China’s 30 provinces over the period 1995–2011, and then analysing the spatiotemporal characteristics. We proceed to examine the key influencing factors quantitatively using panel data models. Based on the coefficients calculated in this study, and taking China as an example, this paper sets up 10 different scenarios in order to forecast future CO_2_ emissions. Under a CO_2_ emissions constraint force, we attempt to identify the optimal development model for China.

The remainder of this study is organised as follows. Section 2 focuses on methods and data, presenting the models and the data utilized. Section 3 displays the results of the paper and sets out a discussion of the scenarios through which the optimal development model was selected. Finally, Section 4 summaries the main conclusions and policy implications.

## Methodology and Data

### Estimating CO_2_ emissions

In this study, CO_2_ emissions are described in terms of CO_2_ equivalent emissions, a measure which refers to the carbon content of greenhouse gases with the same global warming potential. Currently, in China it is very difficult to acquire provincial data for CO_2_ emissions directly. However, because CO_2_ emissions are mainly released through fossil fuel combustion and cement production, these two measures prove useful in approximating CO_2_ emissions through energy-related statistical data. Based on methods developed in previous studies [33], this paper calculates CO_2_ emissions on the basis of these two aspects (that is, the burning of fossil fuel and the cement production process) for China’s 30 provinces from 1995 to 2011. Using the CO_2_ emissions coefficients published by the 2006 the Intergovernmental Panel on Climate Change (IPCC) [50] and the National Coordination Committee Office on Climate Change and the Energy Research Institute under the National Development and Reform Commission [51], the CO_2_ emissions from fossil fuel can be calculated through the following formula:

$$CE=\sum_{i=I}^{7}CE_{i}=\sum_{i=I}^{7}PEC_{i}\times F_{i}$$ (1)

where *CE* denotes the total CO_2_ emissions from energy consumption; *i* represents the types of fossil fuel; *PEC*_*i*_ denotes the consumption quantity of fossil fuel *i*; and *F*_*i*_ is the CO_2_ emissions coefficient of fossil fuels *i* (Table 1).

**Table 1:** CO2 emissions coefficients.

Source	Coal	Coke	Gasoline	Kerosene	Diesel	Fuel oil	Natural gas	Cement
CO_2_ emissions coefficient	1.647	2.848	3.045	3.174	3.150	3.064	21.670	0.527

Sources: IPCC (2006)

CO_2_ emissions from cement production can be calculated using:

CC=Q×F (2)

where *CC* denotes the CO_2_ emissions from the production of cement; *Q* represents the quantity of cement production; and *F* is the CO_2_ emissions coefficient of the cement production process (Table 1).

#### Panel data analysis

Typically, panel data has two dimensions—time and cross-sections. However, compared to the cross-sectional or time series data models, panel data models have several advantages. Firstly, they can expand the amount of information and increase the degree of freedom of estimation and test statistics. Secondly, they can improve the effectiveness of the estimation. Thirdly, effects can be more efficiently recognised and measured than through other models. Fourth, panel data models help to provide the reliability of dynamic analysis. Fifth, they help to detect gradual change in a data structure and institution.

To explore the constraint effect of CO_2_ emissions on the economic development process, a CO_2_ emissions model was developed. Based on existing studies, four variables—namely, population, per capita GDP, energy intensity, and the urbanization level—were utilised as the determinants of CO_2_ emissions. The CO_2_ emissions model is established as follows:

$$\text{ln}EM_{it}=\text{ln}\alpha_{it}+\beta_{1}\text{ln}P_{it}+\beta_{2}\text{ln}A_{it}+\beta_{3}\text{ln}T_{it}+\beta_{4}\text{ln}U_{it}$$ (3)

where ln is a natural logarithm; *EM* denotes CO_2_ emissions; *P* represents the resident population; *A* denotes per capita GDP; *T* represents technological level (energy intensity); *U* is the urbanization level; *β*_1_, *β*_2_, *β*_3_ and *β*_3_ are slope coefficients; *t* denotes time; and *i* denotes the cross-section (provinces).

#### Panel unit root tests

It is well known that panel unit root tests have higher ability than unit root tests that are based on univariate time series or cross section data. This paper will introduce three types of panel unit root test—namely, the Levin-Lin-Chu (LLC) test [52], the Breitung test [53] and the Im-Pesaran-Shin (IPS) test [54]. These tests are commonly utilised because of their high power in stationary tests among variables. The LLC and Breitung tests are widely used in common root tests. The IPS test is widely used in individual root tests.

#### Panel cointegration tests

Compared to normal time series cointegration, panel cointegration tests are widely used among researchers because of their high power [34]. As such, a Pedroni cointegration test, first developed by Pedroni [55], was introduced into the study to examine whether a cointegration relationship existed between the indicators. The Pedroni cointegration test allows for intercepts and trends with different individual effects, and this test is used for both balanced and unbalanced panel data. Compared to traditional panel data models, the Pedroni cointegration test includes a number of great improvements. Specifically, the Pedroni test allows for major differences between the slope coefficient, the fixed effect coefficient and the trend coefficient of different individuals.

Working under a series of assumptions, Pedroni [55] develops two types of residual-based tests. In the first type, four tests are distributed as being standard normal asymptotically and are based on average test statistics for no cointegration in the time series across the cross-section for the within-group [56]. The tests that make up this group are: panel v-statistics, panel ρ-statistics, panel t-Statistic (non-parametric) and panel t-Statistic (parametric). Within the second type, three tests—group r-statistics, group t-statistics (non-parametric) and the group statistics (parametric)–are also distributed as being standard normal asymptotically but are based on limits of piecewise numerator and denominator terms for the limiting distributions for the between-group [56]. Three of the four tests in the first type of test are based on the non-parametric correction test proposed by Phillips and Perron [57]; the remaining test follows the ADF parametric test. Two of the three second-type tests are based on non-parametric tests, and the third test is based on an ADF test.

#### Panel data model

If variables are cointegrated, the Fully Modified OLS (FMOLS) estimator can be utilised to estimate each equation to find out the directions and magnitudes of the influences of the explanatory variables (in this study, population scale, economic level, energy intensity and urbanization level) on CO_2_ emissions. FMOLS, proposed by Pedroni [58], is a single equation estimator for cointegrated relationships. FMOLS uses errors to calculate the cumulative test volume. This approach has been widely used to deal with both temporal and cross sectional dimensions data, as a result of its ability to properly deal with the long-run correlation problem between the cointegrating equation and stochastic regressor innovations [29].

### Scenario simulations

Previous studies have found population, economic level, urbanization and energy intensity to have a significant impact on CO_2_ emissions. Using scientific analysis and a range of different scenarios, relevant existing literature has thus also attempted to identify which scenario might be most conducive to the mitigation of CO_2_ emissions. The identification of such a scenario will have great significance and meaning for the formulation of future economic development policy and the mitigation of CO_2_ emissions. Taking China as an example, in this study we attempted to find the optimal development model for China, from 10 different scenarios which we developed. We assumed that the 10 different scenarios share various evolution paths, so that we might focus on analysing differences in the various development patterns.

#### Scenario description

(1) Economic growth scenario: Table 2 reviews the 3 economic growth scenarios. The “business-as-usual” scenario (B) assumes that the development policy on economic growth stays at the same level as in the Chinese government’s 12th Five-Year Plan (2011–2015) within the forecasting horizon. According to the development experience of developed countries, after rapid economic growth, subsequent economic growth is likely to flatten. Thus, the economic growth rate of the “high economic growth” scenario and the “low economic growth” scenario increase and decrease by 1% respectively based on the economic growth rate in the business-as-usual scenario.

**Table 2:** The economic growth rate of China in the following 10 years.

Scenarios	2010–2015	2015–2020	2020–2025
Business-as-usual scenario	7.5%	7%	6%
Low economic growth scenario	6.5%	6%	5%
High economic growth scenario	8.5%	8%	7%

(2) Population scenario: According to the U.S. Energy Information Administration’s (EIA) [59] forecast and the Chinese government’s 12th Five-Year Plan (2011–2015), the population of China will be controlled to 1.39 billion by 2015 and 1.42 billion by 2020 respectively. Given the above, the average population growth rate will be 0.5% from 2016 to 2020. According to this rate, we therefore can predict that the population of China in 2025 will be 1.45 billion. In the “high population” scenario, the population growth rate increases by 0.1%. Table 3 reviews the predicted population of China in the following 10 years.

**Table 3:** The population of China in the following 10 years (billion).

Scenarios	2015	2020	2025
Middle population growth	1.39	1.42	1.45
High population growth	1.40	1.43	1.47

(3) Urbanization scenario: China has made great success in urbanization, with the urbanization level increasing from 17.9% in 1978 to 51.27% in 2011, at an average annual growth rate of 1.01% [1]. According to the National New-type Urbanization Plan for China released in 2014, the country’s urbanization level will rise by 1% annually, reaching 60% by 2020. Taking the Chinese government’s 12th Five-Year Plan (2011–2015) into consideration, we assumed that the urbanization level in 2015, 2020, and 2025 would be 55%, 60%, and 65% respectively. The urbanization level in the “low urbanization” scenario will decrease by 2% correspondingly. Table 4 reviews predicted levels of urbanization of China for the following 10 years.

**Table 4:** The urbanization of China in the following 10 years.

Scenarios	2015	2020	2025
Middle urbanization development	55%	60%	65%
Low urbanization development	53%	58%	63%

(4) Technological progress scenario: In the content of the 11th Five-Year Plan (2005–2010), China set its goal of reducing energy intensity by 20%. Through the 12th Five-Year Plan (2011–2015), the government plans to make a decrease of 16% in energy intensity [1]. Specifically, energy intensity is to decrease at an average annual rate of 3%. Thus we referred to the targets set out in these plans as constituting the basis for our “middle technology” scenario and assumed an additional 0.5% improvement under the “high technology” scenario. Table 5 reviews China’s predicted technological progress in the following 10 years.

**Table 5:** The technological progress of China in the following 10 years.

Scenarios	2010–2015	2015–2020	2020–2025
Middle technological progress	2.8%	3%	3.5%
High technological progress	3%	3.5%	4%

Based on above analysis, together with the actual development of China in recent years, we set up 10 different scenarios (Table 6). As setting up the 10 scenarios, we consider not only the permutations and combinations of the variables, but also the actual situation of China’s development. Since the goal of this study is to find out the optimal development model for china’s socioeconomic sustainable development and a reduction in CO_2_ emissions. The requires of the best model are not only the lowest CO_2_ emissions, but maintaining relatively high economic growth. Thus, the four variables (economic growth, population growth, urbanization development, and technological progress) combined 10 development scenarios (Scenario B, Scenario BTU, Scenario BH, Scenario L, Scenario LT, Scenario LH, Scenario H, Scenario HP, Scenario HT, and Scenario AH). The detailed content is listed in Table 6.

**Table 6:** The future developing scenarios of China and the descriptions.

Scenarios	Economic growth (*A*)	Population growth (*P*)	Urbanization development (*U*)	Technological progress (*T*)
Scenario B (business as usual)	Business as usual	Middle	Middle	Middle
Scenario BTU (business as usual, high technology, low urbanization)	Business as usual	Middle	Low	High
Scenario BH (business as usual, high technology, high population)	Business as usual	High	Middle	High
Scenario L (low economic growth)	Low growth	Middle	Low	Middle
Scenario LT (low economic growth, high technology)	Low growth	Middle	Low	High
Scenario LH (business as usual, high technology, high population, high urbanization)	Low growth	High	Middle	High
Scenario H (high economic growth)	High growth	Middle	Middle	Middle
Scenario HP (high economic growth, high population)	High growth	High	Middle	Middle
Scenario HT (high economic growth, high technology)	High growth	Middle	Middle	High
Scenario AH (all high growth)	High growth	High	Middle	High

Note: “Low” denotes the low scenario, “Middle” denotes the middle scenario, and “High” denotes the high scenario.

#### Methods for forecasting future CO_2_ emissions

According to the results of the panel data analysis, the following regression model will be utilized to forecast CO_2_ emissions for each province under 10 different scenarios for the year 2015 to 2025:

$$\hat{\text{ln}EM_{it}}=\hat{\alpha }+{\hat{\beta }}_{1}\text{ln}P_{it}+{\hat{\beta }}_{2}\text{ln}A_{it}+{\hat{\beta }}_{3}\text{ln}T_{it}+{\hat{\beta }}_{4}\text{ln}U_{it}\text{+}{\hat{\eta }}_{i}$$ (4)

where *EM* denotes the total CO_2_ emissions both form the burning of fossil fuel and the cement production process.

The following exponential transformation will be further implemented since we aim to forecast *EM*_*it*_:

$$\hat{EM_{it}}=\text{exp}\left(\hat{\alpha }+{\hat{\beta }}_{1}\text{ln}P_{it}+{\hat{\beta }}_{2}\text{ln}A_{it}+{\hat{\beta }}_{3}\text{ln}T_{it}+{\hat{\beta }}_{4}\text{ln}U_{it}\text{+}{\hat{\eta }}_{i}\right)$$ (5)

Once each province’s CO_2_ emissions are estimated, we then calculate the emissions of China using:

$$\hat{EM_{t}}=\sum_{i=1}^{30}\hat{EM_{it}}t=2015,\;2020,\;2025$$ (6)

### Data

**Fig 1:**
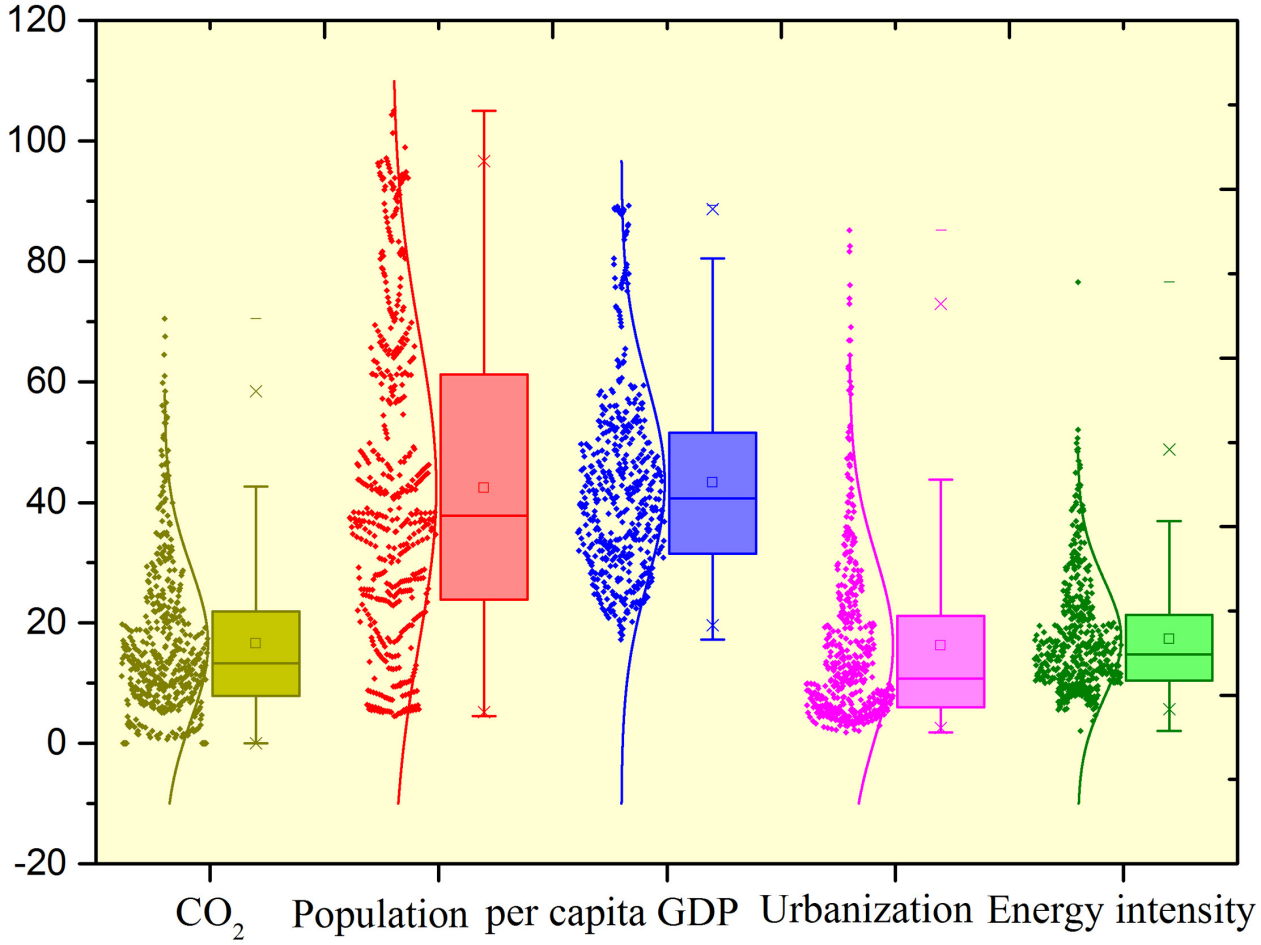
The distributions of the variables in box-chart form, for China’s 30 provinces, 1995–2011.

Based on the availability of data, this study used annual time series data for 30 provinces in China, in the period 1995–2011. Taiwan and Tibet were excluded on the basis of missing data for most of the years. Provincial data on resident populations, per capita GDP, cement, energy intensity and urbanization levels were derived from the China Statistical Yearbook and provincial statistical yearbooks. To eliminate the price effect, per capita GDP data were adjusted by considering the official price index in the year 2000, which was also used to calculate energy intensity in tons per 10^4^ Yuan. All the data on fossil fuel were derived from the China Energy Statistical Yearbook. The total energy consumption and each fossil fuel were all converted into standard coal measures (units of 10^4^ tons). Annual data on CO_2_ emissions for each province were estimated using (Eq 1) and (Eq 2). Table 7 displays the statistical description of the five variables in the 30 Chinese provinces, covering the years 1995 to 2011. The distribution parameters of the five variables in China’s 30 provinces are presented as box and whisker plots with the bottom and top of the box representing the 25th and 75th centiles (Fig 1).

**Table 7:** Summary statistics of the variables.

Variables	Symbol	Unit	Mean	Std. Dev	Min	Max
CO_2_ emissions	*EM*	10^4^tons	16830.07	12448.91	680.56	70542.8
Population	*P*	10^4^ persons	4242.92	2576.69	456.2	10505
Per capita GDP	*A*	Yuan	16224.63	14829.06	1853	85213
Energy intensity	*T*	ton/10^4^ Yuan	1.73	0.98	0.21	7.66
Urbanization	*U*	%	43.33	16.40	17.19	89.30

## Results and Discussion

### Spatiotemporal characteristics of provincial CO_2_ emissions

**Fig 2:**
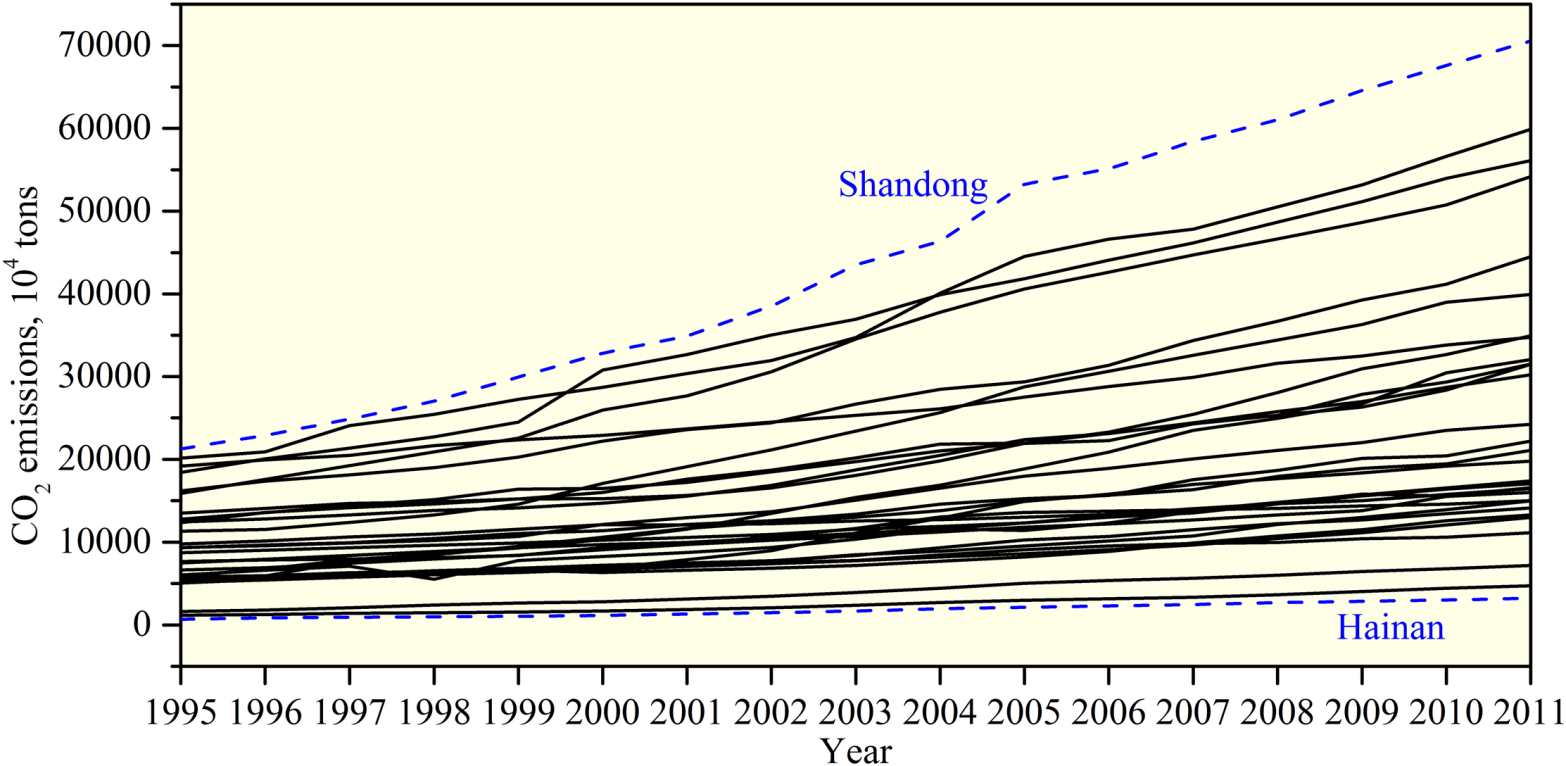
Growth trajectories of CO_2_ emissions in provincial China from 1995 to 2011.

**Fig 3:**
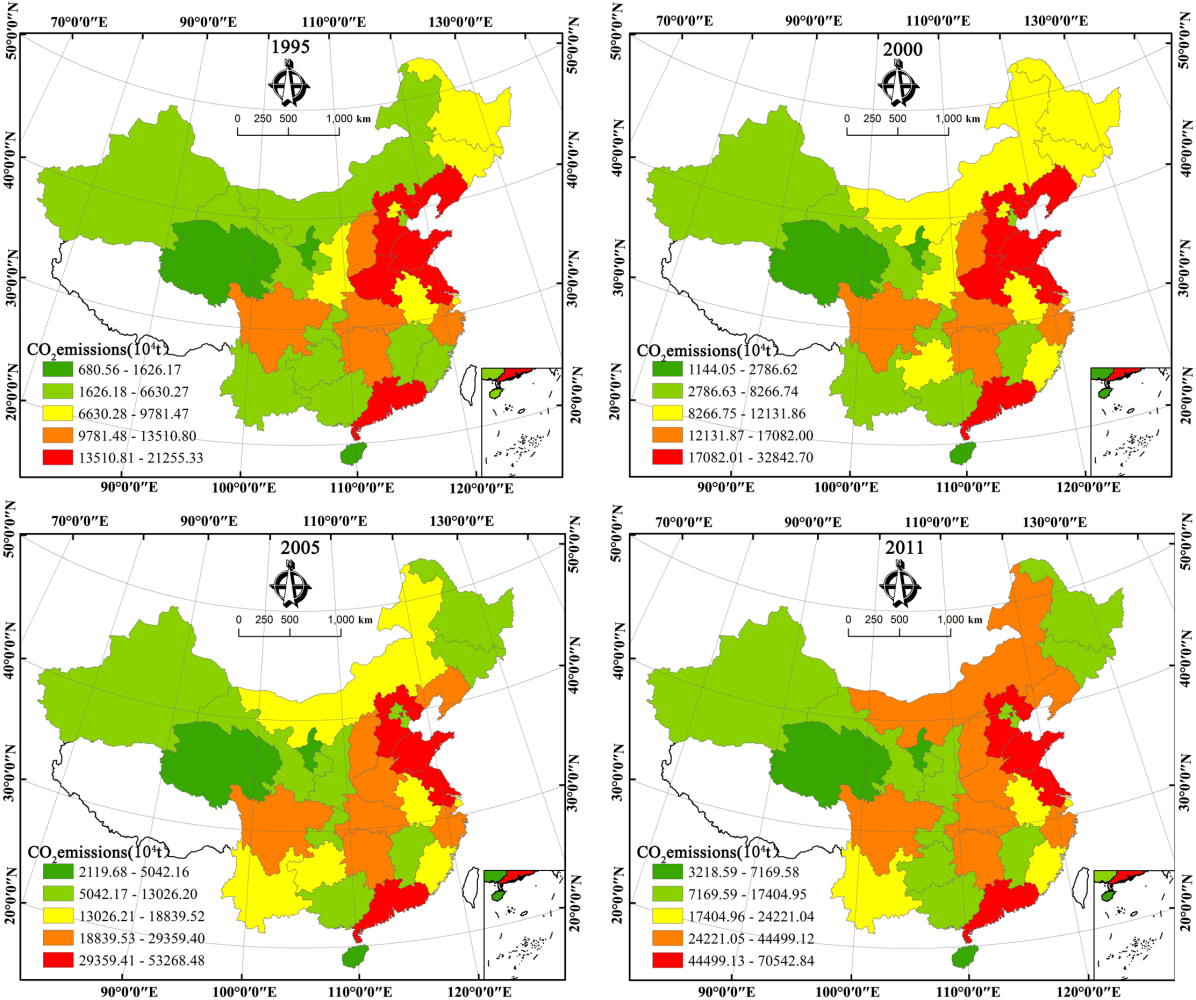
Spatial distributions of CO_2_ emissions for selected years.

Once the emissions of each province were estimated from 1995 to 2011, it is necessary to analyse the spatiotemporal characteristics in emissions. Fig 2 provides a general overview of the growth of CO_2_ emissions in provincial China from 1995 to 2011. From Fig 2, we find that CO_2_ emissions for each province increased over time. During the study period, Shandong Province was the largest emitter with its emissions rising from 212.55 million tons in 1995 to 705.42 million tons in 2011, while Hainan Province was the smallest emitter with its emissions increasing from 6.81 million tons in 1995 to 32.18 million tons in 2011. The former province produces more CO_2_ emissions owing to its huge consumption of fossil energy compared to other regions. Notably, CO_2_ emissions form east regions were initially larger and increased more rapidly than their neighbours in central and west regions during the period studied. From the viewpoint of geography, CO_2_ emissions in China exerted noticeable regional discrepancies in 1995–2011 (Fig 3). Provinces with high CO_2_ emissions were found to be mainly concentrated in eastern coastal China; in fact, CO_2_ emissions decrease gradually from the eastern coastal region to the central region, and then to the western region.

**Fig 4:**
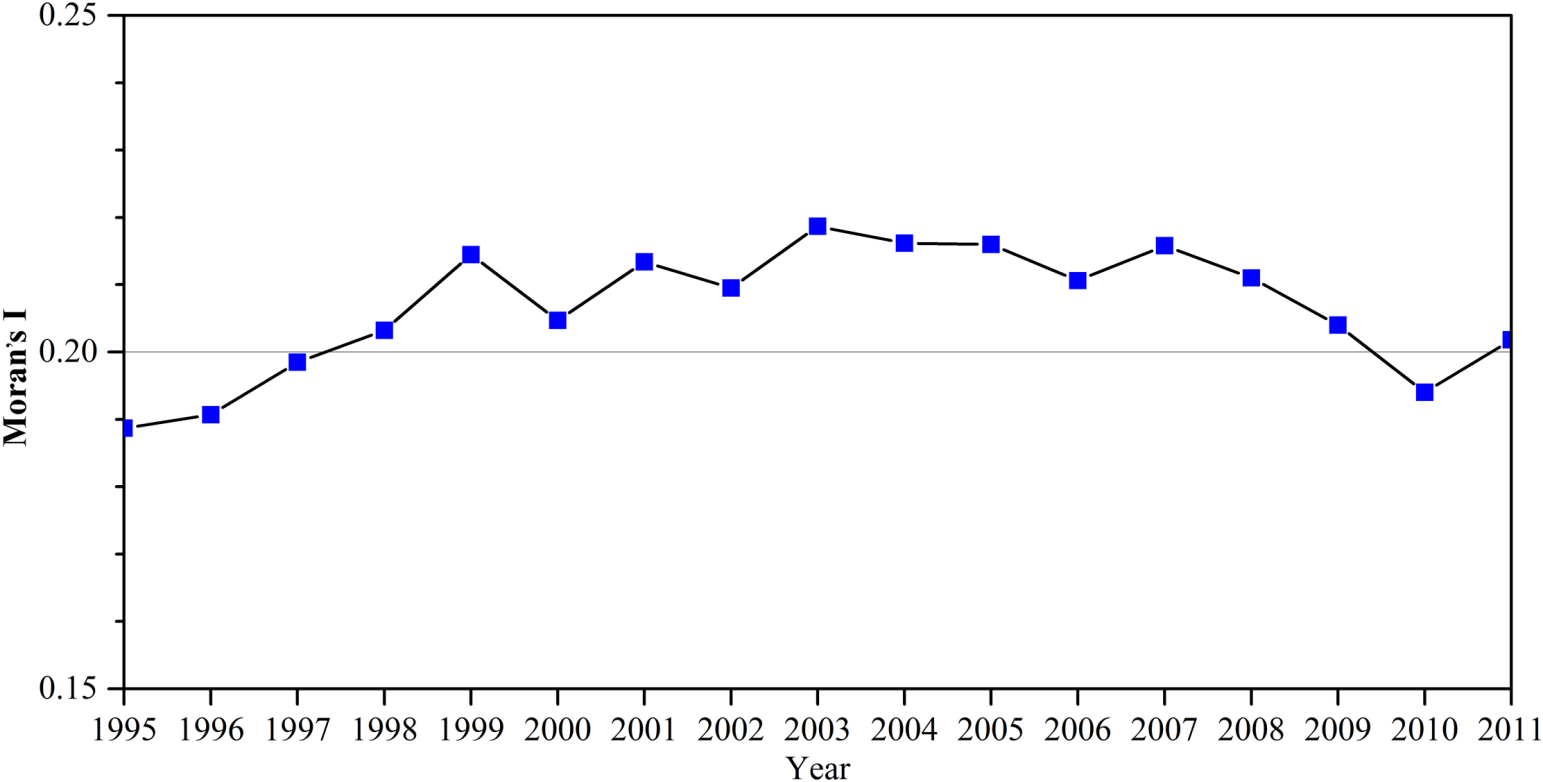
Moran’s I for regional CO_2_ emissions in provincial China from 1995 to 2011.

**Fig 5:**
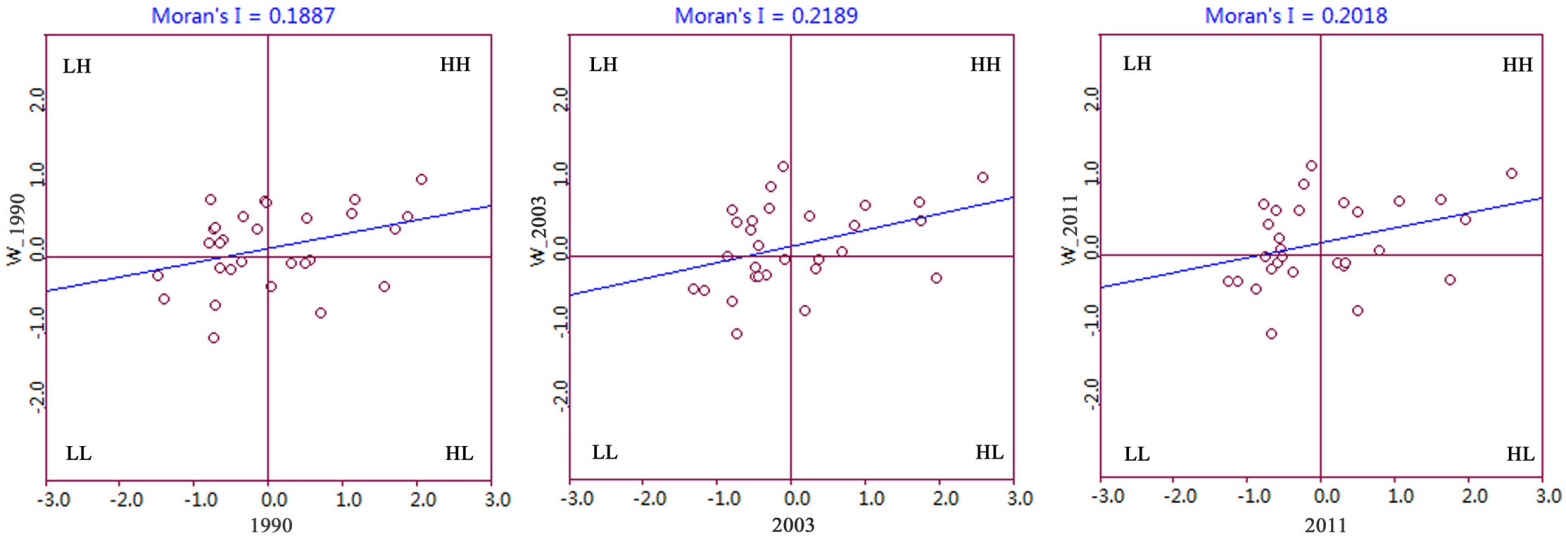
Moran scatter plot of CO_2_ emissions for selected years. HH means high values surrounded by high values; LH means low values surrounding by high values; LL means low values surrounded by low values; HL means high values surrounded by low values.

We then calculated global Moran’s I as an index to detect spatial dependence. Fig 4 plots the trajectories of the spatial dependence of CO_2_ emissions in provincial China from 1995 to 2011. As indicated in Fig 4, Moran’s I fluctuates around 0.2, all are significant at 95% confidence level over the period studied. This finding provided evidence of spatial dependence taking place in provincial China. In addition, the fluctuations of Moran’s I reflect the temporal dynamics of the spatial dependence of CO_2_ emissions, which may weaken or strengthen the agglomeration of CO_2_ emissions over time. From the viewpoint of scatter distribution (Fig 5), HH and LL clusters constitute the main types of spatial dependence seen over the studied period.

**Fig 6:**
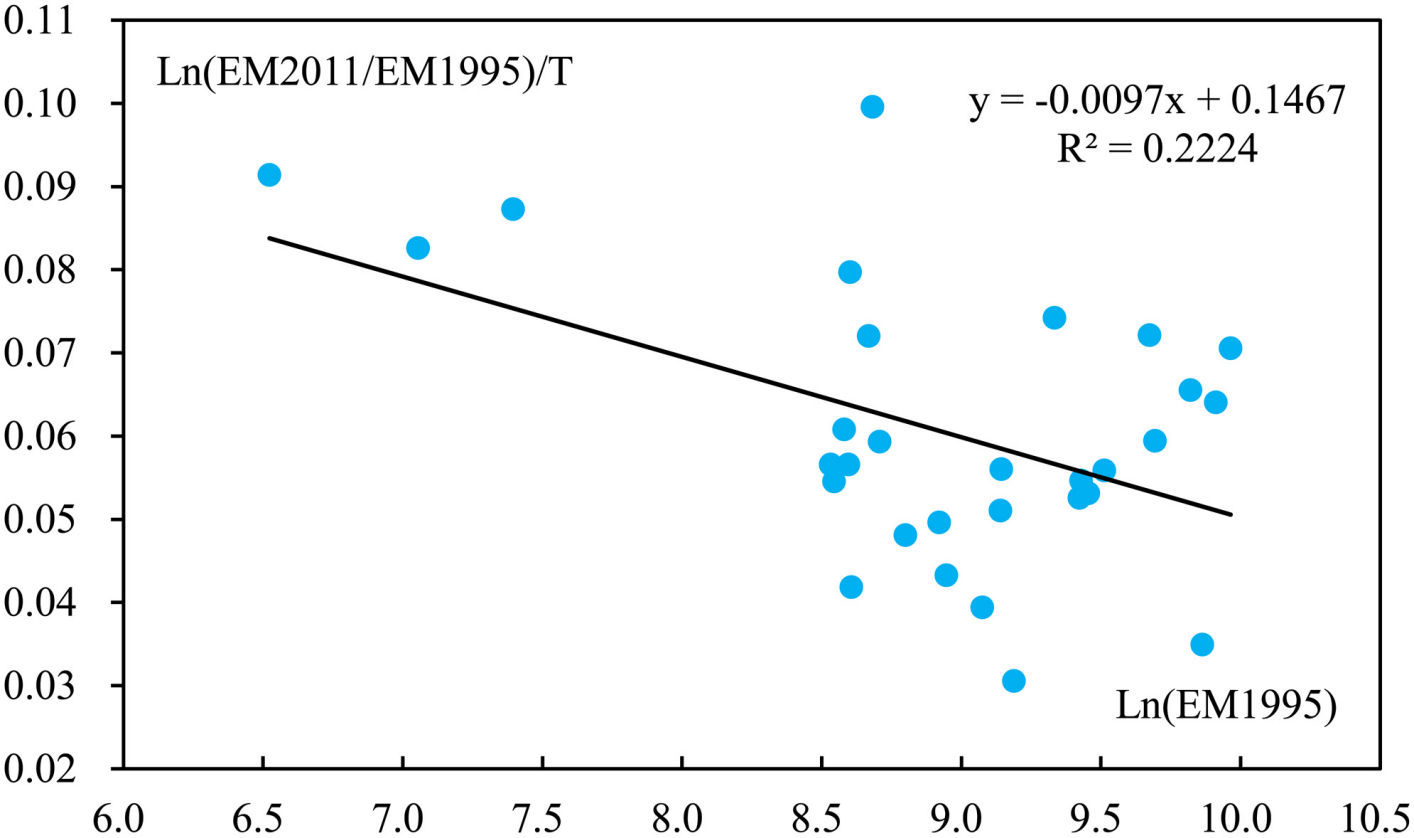
Annual growth rate and level of CO_2_ emissions. Ln denotes the nature log; EM represents CO_2_ emissions; T denotes year.

Fig 6 provides an analysis of the emission level in 1995 and annual growth rate during the period studies. An estimated linear regression model (R^2^ = 0.2224) is also displayed in Fig 6. The negative slope of the linear regression equation implies a convergence trend in CO_2_ emissions in Chinese provinces.

### Influencing factors of provincial CO_2_ emissions

#### Results of panel unit root tests

The analysis of casual relationships between variables has been the hot topic in modern panel data analysis. However, not all variables have cointegrating relationships. Thus, prior to cointegration estimation, it is necessary to test the stationarity of variables; this is known as a unit root test. In this study, we employed three types of panel unit root test: the Levin-Lin-Chu (LLC) test, the Breitung test and the Im-Pesaran-Shin (IPS) test. The LLC and Breitung methods are both suitable for common root tests, and the IPS method is suitable for individual root tests. Table 8 reviews the results of the panel unit root test for both types. As indicated by Table 8, the results clearly show that all the variables cannot reject the null hypothesis at level, indicating that they were not stationary at level. However, all the variables were found to be stationary at the first difference, rejecting the null hypothesis. As such, we were able to carry out a Pedroni cointegration test in order to estimate whether a cointegrating relationship existed between the variables. If the variables were found to be cointegrated, the Fully Modified OLS estimator would be used to examine the relationship quantitatively.

**Table 8:** Panel unit root test results.

Variable	Level		First difference	
	Intercept	Intercept and trend	Intercept	Intercept and trend
Levin-Lin-Chu test (common root)				
ln*EM*	9.86438	11.8364	-21.2607 ^a^	-19.1668 ^a^
ln*P*	7.45089	3.37603	-12.1889 ^a^	-13.6134 ^a^
ln*A*	12.8813	0.89036	-5.85314 ^a^	-11.7708 ^a^
ln*T*	4.21049	7.39003	-15.8270 ^a^	-14.3800 ^a^
ln*U*	7.11117	-0.14841	-12.1172 ^a^	-12.4863 ^a^
Breitung test (common root)				
ln*EM*		-4.16615 ^c^		-8.94631 ^b^
ln*P*		0.93245		-7.29138 ^b^
ln*A*		7.20825		-5.48287 ^a^
ln*T*		-1.92443 ^b^		-2.07202 ^a^
ln*U*		-1.38384 ^c^		-6.69167 ^a^
Im-Pesaran-Shin test (individual root)				
ln*EM*	6.94940	5.96649	-17.0560 ^a^	-13.5340 ^a^
ln*P*	0.43313	0.65222	-10.3251 ^a^	-8.87672 ^a^
ln*A*	19.7374	7.53347	-3.75383 ^a^	-8.69289 ^a^
ln*T*	2.02629	-5.47261 ^b^	-15.0668 ^a^	-11.1620 ^a^
ln*U*	2.31694	3.11780	-9.20053 ^a^	-8.95612 ^a^

Note: the unit root tests were carried out with individual trends and intercepts for each variable, and the optimal lag lengths were selected automatically using the Schwarz information criteria.

^a^ Denotes significance at the 1% level.

^b^ Denotes significance at the 5% level.

^c^ Denotes significance at the 10% level.

#### Panel cointegration results

Since all the variables used in this study were stationary at their first difference, we proceeded to test the dependent and independent variables respectively for Pedroni cointegration in the data, using the heterogeneous panel cointegration test first proposed by Pedroni [55]. Table 9 presents the panel cointegration test results. To examine the cointegrating relationship more accurately, we employed three trend assumptions from the Pedroni cointegration test, namely: no deterministic trend, deterministic intercept and trend, and no deterministic intercept or trend. From the results detailed in Table 9, we found that under the assumption of no deterministic trend, six statistics strongly rejected the null hypothesis of no-cointegration significantly at the 1% level; while under the assumption of deterministic intercept and trend, seven statistics were found to reject the null hypothesis of no cointegration at less than the 10% level. In addition, under the assumption of no deterministic intercept or trend, six statistics also strongly rejected the null hypothesis of no cointegration significantly at less than the 5% level. Thus we obtained clear evidence of the existence of a cointegrating relationship between the variables. Results also indicate that population scale, economic level, energy intensity and urbanization level had a long-run relationship with CO_2_ emissions in China’s 30 provinces during the study period. The next step was to estimate this relationship using Fully Modified OLS regression.

**Table 9:** Pedroni cointegration test results.

Trend assumption	No deterministic trend	Deterministic intercept & trend	No deterministic intercept or trend
Alternative hypothesis: common AR coefficients (within-dimension)			
Panel v-statistic	-0.265419	-0.238256	-1.525700
Panel rho-statistic	2.603084	4.425116	1.319566
Panel PP-statistic	-2.458144 ^a^	-2.526636 ^a^	-3.103253 ^b^
Panel ADF-statistic	-3.627780 ^a^	-1.493838 ^c^	-3.694388 ^a^
Panel v-statistic (weighted)	-0.191670	-0.150265 ^c^	-1.649109
Panel rho-statistic (weighted)	2.611259	4.579338	1.895627
Panel PP-statistic (weighted)	-2.902991 ^a^	-2.352576 ^a^	-1.779753 ^a^
Panel ADF-statistic (weighted)	-4.649224 ^a^	-2.532676 ^a^	-2.592808 ^a^
Alternative hypothesis: individual AR coefficients (between-dimension)			
Group rho-statistic	4.931659	6.604612	4.056057
Group PP-statistic	-4.383673 ^a^	-2.439537 ^a^	-2.698744 ^a^
Group ADF-statistic	-4.604860 ^a^	-2.321178 ^b^	-4.429429 ^a^

Note: The optimal lag length was automatically selected based on the Schwarz. Newey-West automatic bandwidth was selected based on a Bartlett kernel.

^a^ Denotes significance at 1% level.

^b^ Denotes significance at 5% level.

^c^ Denotes significance at 10% level.

#### Parameter estimations of the panel model

**Fig 7:**
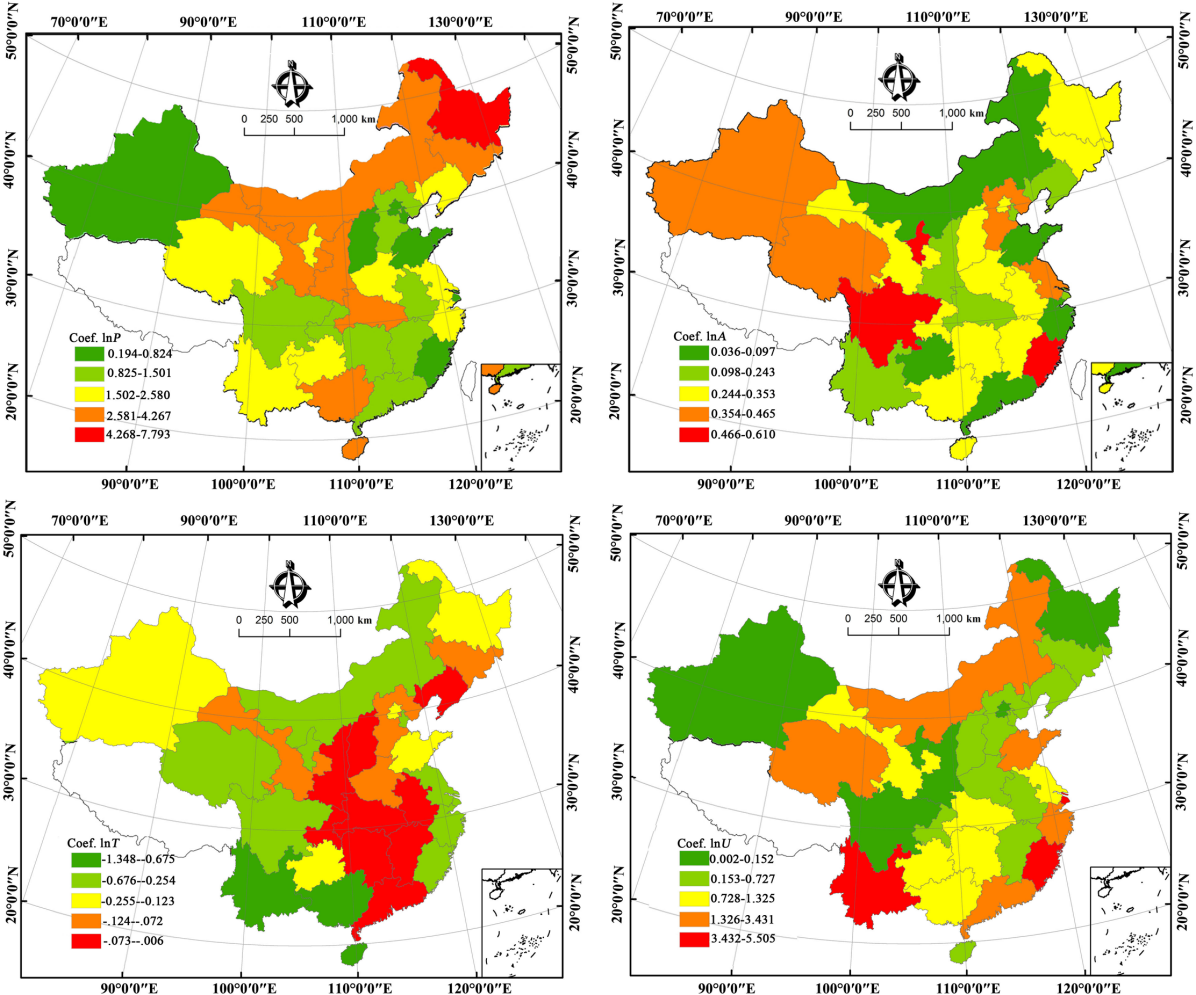
The spatial distributions of coefficients of the independent variables for China’s 30 provinces, 1995–2011. Coef. (lnP, lnA, lnT, lnU) denote the coefficients of lnP, lnA, lnT, and lnU.

Since the variables used in this study were cointegrated, we proceeded to estimate the relation. A Fully Modified OLS test was utilised based on provincial panel data. A panel data model with a Newey-West fixed effect was adopted; the Kernel option of the model was based on the Bartlett law. Table 10 reviews the results of the Fully Modified OLS test. Parameter estimations of the panel data model reveal that the individual variable coefficients have important but various influence on CO_2_ emissions. Specifically, population scale, economic level and urbanization level have positive effects on CO_2_ emissions, while energy intensity produces negative, inhibitory effects. First, as expected, the increase of population directly leads to an increasing demand of energy resources and, in turn, produce a large amount of CO_2_ emissions. Second, since the Chinese economic reform in 1978, China has witnessed rapid economic growth. However, this rapid growth has been achieved by consuming a large amount of energy resources, leading to a great deal of CO_2_ emissions. Third, millions of people lived in rural areas moved to cities and towns across China every year. This migration creates increasing pressure on the country's urban sustainable development. Urbanization has a promoting effect on economic development, but it can also contribute to the increase of energy consumption. Finally, reducing energy intensity (per capita GDP energy consumption) is conducive to reduce CO_2_ emissions. That is because improving energy efficiency mean producing the same amount of GDP with mitigated energy use, which will indirectly migitate CO_2_ emissions [18]. In addition, the significance of the relationship between CO_2_ emissions and the four variables also varied across the provinces based on their scale of social and economic development. Similar results were found by Al-mulali [34] in the Middle East and Li et al. [56] at regional and national levels in China. From the perspective of geographic analysis (Fig 7), provinces with high coefficients of population and energy intensity were found to be mainly concentrated in northern and central China respectively. However, areas with relative high coefficients of the other two variables (per capita GDP and urbanization) were found to have decentralized distributions. Since this study used a panel data model, we will focus on the panel effect and panel analysis results in this paper. The panel FMOLS test results show that all the four variables—population, per capita GDP, energy intensity and urbanization—all maintained a positive long-run relationship with CO_2_ emissions—that is: a 1% increase in total population will increase CO_2_ emissions by 1.359500% and a 1% increase in per capita GDP will increase CO_2_ emissions by 0.373704%, and, in addition, a 1% increase in energy intensity will increase CO_2_ emission by 0.277178%. Finally, a 1% increase in urbanization will increase CO_2_ emission by 0.591743%. The results of the study indicate that population, per capita GDP, energy intensity and urbanization are the main human factors that influence CO_2_ emissions in the long run. These results are supported by studies undertaken by Wang et al. [18] in Beijing city, Wang et al. [20] Guangdong province, Al-mulali [34] in Middle East, Soytas et al. [60] in the United States, Hamit-Haggar [61] in Canada, Niu et al. [62] in 8 Asian-Pacific countries, Pao and Tsai [63] in BRIC countries, and Kum et al. [64] in G-7 countries.

**Table 10:** Fully modified OLS test results.

Province	ln*EM* is the dependent variable			
	ln*P*	ln*A*	ln*T*	ln*U*
Beijing	0.824183 (11.30692) ^a^	0.289275 (0.876098) ^c^	-0.131756 (-1.333606) ^c^	-0.152019 (-0.468544) ^c^
Tianjin	0.694356 (4.258218) ^a^	0.171245 (2.248154) ^b^	-0.254462 (-2.58327) ^b^	-0.300521 (-0.492524) ^c^
Hebei	1.500945 (-0.612880) ^c^	0.387984 (2.301110) ^b^	-0.072337 (-0.412595) ^c^	0.602407 (2.481098) ^a^
Shanxi	0.551093 (0.959887) ^c^	0.301567 (4.208939) ^a^	0.049979 (0.521487) ^c^	0.541600 (2.967801) ^b^
Inner Mongolia	3.277176 (1.191737) ^c^	0.036837 (0.239765) ^c^	0.577542 (3.538200) ^a^	3.431111 (1.865341) ^c^
Liaoning	1.845928 (0.890808)	0.173370 (2.402487) ^b^	0.005342 (0.587733) ^c^	0.375841 (4.107257) ^a^
Jilin	4.158499 (3.100181) ^b^	0.321985 (21.05442) ^a^	0.100307 (2.468636) ^b^	-0.726538 (-2.130747)*
Heilongjiang	7.793006 (3.701333) ^a^	0.334755 (2.814689) ^b^	0.179252 (0.994231)	0.758633 (1.679754) ^c^
Shanghai	0.736489 (3.443010) ^a^	0.199758 (1.771537) ^c^	-0.319790 (-2.034266) ^c^	4.801402 (1.974920) ^c^
Jiangsu	1.972789 (-0.750660)	0.454410 (1.710387) ^c^	0.331841 (1.938560) ^c^	1.324628 (3.837937) ^a^
Zhejiang	2.407720 (6.934410) ^a^	0.095659 (1.908148) ^c^	0.290727 (4.341431) ^a^	2.492042 (16.88751) ^a^
Anhui	0.993859 (1.378247) ^c^	0.326869 (4.872980) ^a^	0.006397 (0.048812) ^c^	0.309674 (4.702756) ^a^
Fujian	0.614288 (0.261912) ^c^	0.521356 (-4.4651) ^a^	-0.380492 (-2.72180) ^b^	5.504444 (4.492441) ^a^
Jiangxi	1.181380 (0.852470) ^c^	0.352563 (1.986303) ^c^	-0.040064 (-0.435103)	0.721609 (4.703909) ^a^
Shandong	0.214461 (0.200950) ^c^	0.088948 (2.750715) ^b^	0.122598 (3.541862) ^a^	2.252716 (15.74788) ^a^
Henan	2.091465 (4.251184) ^a^	0.293459 (4.791035) ^a^	-0.092854 (-1.355126) ^c^	0.341816 (2.268962) ^b^
Hubei	3.467381 (2.927361) ^b^	0.213272 (4.659398) ^a^	-0.047483 (-0.577729) ^c^	1.000147 (3.967640) ^a^
Hunan	1.053785 (3.175042) ^a^	0.305124 (3.209294) ^a^	0.008492 (0.209965) ^c^	0.945590 (8.912996) ^a^
Guangdong	1.168380 (10.03458) ^a^	0.050295 (-1.313565) ^c^	-0.045818 (-1.522928) ^c^	2.310187 (7.085244) ^a^
Guangxi	3.915327 (4.343413) ^a^	0.323124 (1.796867) ^c^	0.675417 (3.353234) ^a^	1.140652 (1.956864) ^c^
Hainan	3.024624 (2.562536) ^b^	0.353403 (1.097361) ^c^	0.722216 (1.993635) ^c^	-0.322842 (-1.181016) ^c^
Chongqing	1.280668 (4.016290) ^a^	0.303874 (6.178282) ^a^	0.028903 (0.561329) ^c^	0.572423 (3.760052) ^a^
Sichuan	1.148358 (2.598093) ^a^	0.609136 (14.35764) ^a^	0.283824 (3.893509) ^a^	-0.032401 (-0.232509) ^b^
Guizhou	2.579990 (3.144259) ^a^	0.097241 (0.356331) ^c^	-0.129274 (-0.723764) ^c^	1.304333 (1.040142) ^b^
Yunnan	-6.159211 (-2.163103) ^c^	0.146812 (0.747565) ^b^	1.348419 (4.304103) ^a^	4.021002 (4.038191) ^a^
Shaanxi	4.267050 (3.315569) ^a^	0.243101 (6.012256) ^a^	-0.010848 (-0.435752) ^c^	-0.018400 (-0.058465) ^c^
Gansu	4.126984 (4.754707) ^a^	0.306104 (0.109017) ^c^	-0.093864 (-1.166967) ^c^	0.942129 (5.731639) ^a^
Qinghai	2.147035 (4.759040) ^a^	0.428308 (2.723734) ^b^	0.339483 (2.309805) ^b^	2.738816 (3.855121) ^a^
Ningxia	1.95013 (5.578269) ^a^	0.551342 (-4.00193) ^b^	-0.339291 (-3.29132) ^b^	0.104496 (0.246638) ^c^
Xinjiang	0.194117 (0.302559) ^c^	0.465409 (3.115279) ^a^	-0.168746 (-1.181087) ^c^	0.002431 (0.004111) ^c^
Panel	1.359500 (12.57321) ^a^	0.373704 (15.77366) ^a^	0.277178 (7.872822) ^b^	0.591743 (8.880020) ^a^

^a^ Denotes significance at 1% level.

^b^ Denotes significance at 5% level.

^c^ Denotes significance at 10% level.

### Forecasting CO_2_ emissions

**Fig 8:**
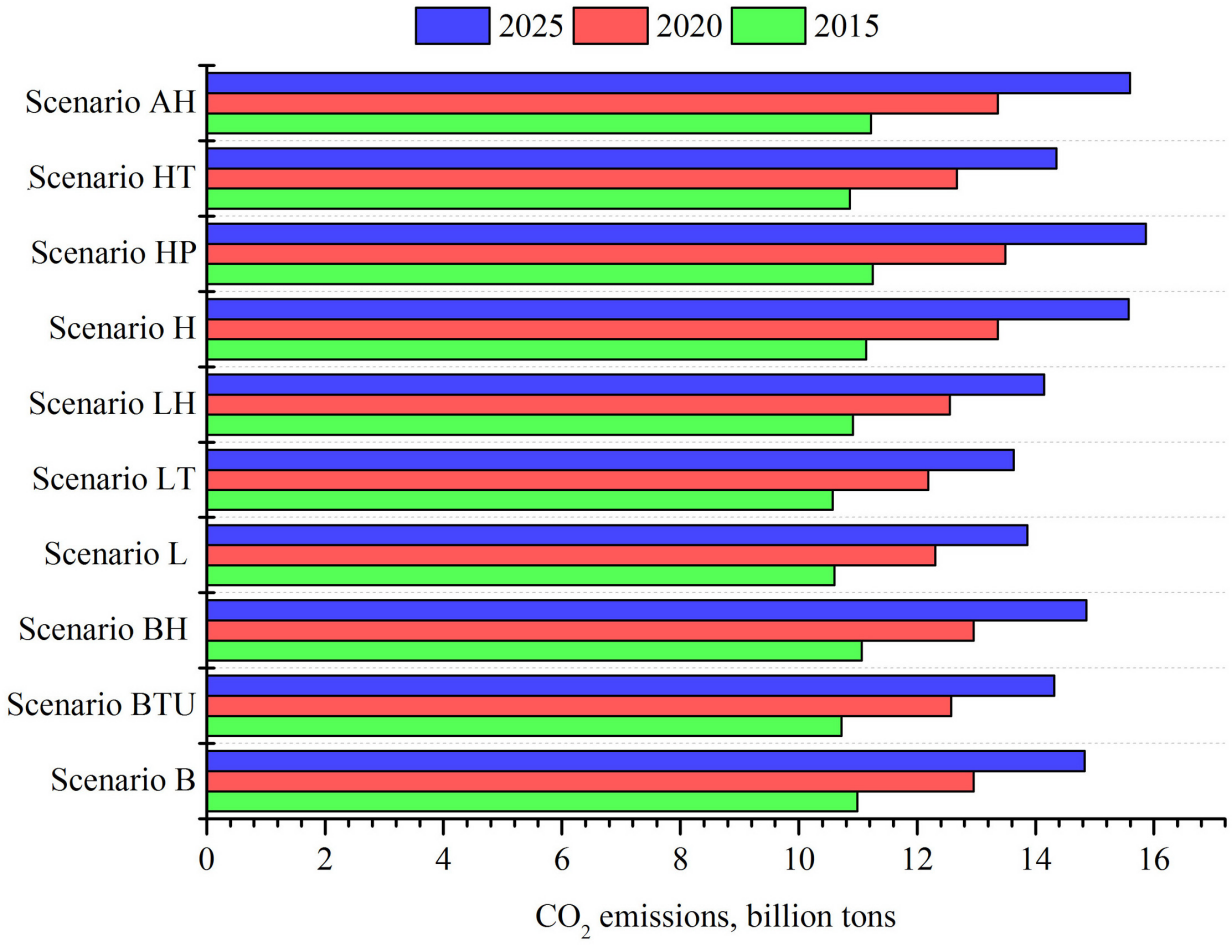
The forecasting results of CO2 emissions under different scenarios, 2015, 2020, 2025.

Fig 8 plots the forecasting results of CO_2_ emissions in 2015, 2020 and 2025 under the scenarios listed at Table 10. The results detailed in Fig 8 reveal that China’s CO_2_ emissions will reach 10.57–11.25, 12.18–13.49, and 13.63–15.60 billion tons by 2015, 2020, and 2025 respectively.

Fig 8 shows that, under Scenario B (business as usual), China’s CO_2_ emissions are expected to reach 10.98, 12.95, and 14.83 billion tons by 2015, 2020, and 2025 respectively. On the basis of the B scenario, if the economic growth rate increases by 1% (Scenario H), China’s CO_2_ emissions will increase by 0.15, 0.41, and 0.74 billion tons in 2015, 2020, and 2025 respectively. With rapid economic growth, if the population growth rate continues to increase (Scenario HP), China’s CO_2_ emissions will increase by 0.26, 0.54, and 1.03 billion tons by 2015, 2020, and 2025 respectively, compared to Scenario B. However, if the development and use of low-carbon technologies could be promoted, and economic development simultaneously boosted (Scenario HT), China’s CO_2_ emissions will decrease by 0.12, 0.28, and 0.42 billion tons by 2015, 2020, and 2025 respectively. If the population continues to increase on the basis of the HT scenario, China’s CO_2_ emissions will increase by 0.23, 0.41, and 0.77 billion tons, in comparison with Scenario B.

The findings of this study indicate that in a scenario where the country’s economic growth rate decreases by 1% and the process of urbanization concurrently slows (Scenario L), China’s CO_2_ emissions will decrease by 0.39, 0.65, and 0.97 billion tons by 2015, 2020, and 2025 respectively, in comparison with Scenario B. If the economic growth rate decreases, however, and low-carbon technologies have been promoted (Scenario LT), China’s CO_2_ emissions will decline by 0.41, 0.77, and 1.20 billion tons by 2015, 2020, 2025 respectively, compared to Scenario B. At the same time, of all the scenarios explored in this study, we find China to have the lowest CO_2_ emissions under Scenario LT. If the country’s population continues to increase on the basis of Scenario LT, CO_2_ emissions will increase accordingly. If the economy has a middle level of growth, urbanization is relatively low, low-carbon technologies rapidly improve, and population growth occurs at a middle level (Scenario BTU), China’s CO_2_ emissions will decrease by 0.27, 0.38, and 0.52 billion tons by 2015, 2020, and 2025 respectively, compared to Scenario B. However, if the population has a high growth rate, as in Scenario BH, China’s CO_2_ emissions will increase accordingly by 0.34, 038, 0.54 billion tons by 2015, 2020, and 2025 (compared to Scenario BTU).

The above analysis indicates that China would have the lowest CO_2_ emissions under Scenario LT. However, Scenario LT requires that the economy maintain a relatively low level of growth, that population growth maintain a middle level, that urbanization increase at a low level, and that low-carbon technologies be promoted widely. The rapid development model currently pursued by China does not conform to these conditions. As the world’s largest CO_2_ emitter, China faces the dual challenge of mitigating CO_2_ emissions while concurrently maintaining economic growth. Therefore, among the 10 scenarios, only the Scenario BTU conforms to the aim of maintaining a middle level of economic growth and effectively reducing CO_2_ emissions.

It is essential to compare the results of our estimations of CO_2_ emissions with other recent studies. Table 11 reviews the results of estimations made by Chen et al. [65], IEA [66], Cai et al. [67], EIA [68], ERI [69], Du et al. [33] and this study. The estimated emissions detailed in this study are higher than that of IEA [66], Cai et al. [67], and EIA [68], but are close to that of Du et al. [33]. It is notable that two sources were considered in estimating CO_2_ emissions in this study: the combustion of fossil fuel and the cement production process. It is noted that the studies identified above only consider fossil fuel related emissions. Du et al. [33], however, accounted for emissions from both the sources, but set scenarios and selected impact factors which varied slightly from those used in the present study. Despite these differences, we note that our forecasting result is quite close to the result obtained by Du et al. [33].

**Table 11:** Compared with other studies estimating China’s CO_2_ emissions.

Literature	Estimating methods	Estimating period	2015 (100 million tons)	2020 (100 million tons)
Chen et al. (2004)	Structural decomposition analysis	2005–2020	—	130–200
IEA (2008)	System optimization	2007–2030	88.28	100.04
Cai et al. (2008)	Bottom-up sector-based analysis	2001–2020	—	53.67
EIA (2009)	System optimization	2015–2035	82.04	94.17
ERI (2009)	System optimization	2005–2050	—	78.54
Du et al. (2012)	Panel data econometrics	2010–2020	113	147.09
This study (2014)	Panel data model	2015–2025	107.2	125.7

Note: the forecasting results taken from Du et al. (2012) are estimated under a business-as-usual scenario. The forecasting results in this study are estimated under Scenario BTU.

## Conclusions and Policy Implications

China’s rapid economic growth has brought about increases in the scale of carbon dioxide (CO_2_) emissions produced by the country. A growing question lies in the optimal development model for China—which model would allow China to face the challenge of mitigating CO_2_ emissions while concurrently maintaining economic growth? In addressing this question, this paper investigated the spatiotemporal variations, influencing factors and scenario simulations of China’s CO_2_ emissions, based on a provincial panel data set covering the period of 1995 to 2011. To examine the influencing factors of CO_2_ emissions quantitatively, a CO_2_ emissions model was developed considering CO_2_ emissions as the dependent variable and population, per capita GDP, energy intensity and the urbanization level as the independent variables. Panel unit root tests, a panel cointegration test, and FMOLS estimator were all utilised. Building on the estimated relationship between CO_2_ emissions and the four independent variables studied, and taking China as an example, this paper created 10 different scenarios to forecast future CO_2_ emissions. Under a CO_2_ emissions constraint force, we attempted to find the optimal development model for China.

There were significant differences of CO_2_ emissions among China’s provinces. CO_2_ emissions of all provinces have increased over the studied period and the emissions varied across the provinces. In addition, evidence of spatial dependence was identified in provincial CO_2_ emissions, and the fluctuations of Moran’s I may have important role in the agglomeration of CO_2_ emissions over time. A graphical analysis of the initial level and average annual growth rate of CO_2_ emissions suggested a convergence existing in provincial China.

Parameter estimations of the panel data model revealed that population scale, economic level and urbanization level positively influenced CO_2_ emissions, while energy intensity exerted negative, inhibitory effects. In addition, the significance of the relationship between CO_2_ emissions and the four variables varied across the provinces based on their scale of social and economic development. From the perspective of panel data analysis, and focusing on panel effect, a quantitative relationship between CO_2_ emissions and the four independent variables was identified: a 1% increase in population, per capita GDP, energy intensity and urbanization was found to result in an increase in CO_2_ emissions by 1.359500%, 0.373704%, 0.277178%, and 0.591743% respectively.

To achieve the goal of curbing CO_2_ emissions while maintaining economic growth, this paper reviewed 10 different developing scenarios. The study forecasted future CO_2_ emissions based on the relationship obtained from the panel data analysis. Under a CO_2_ emissions constraint force, scenario simulations further showed that Scenario BTU—that is, middle economic growth, middle population increase, low urbanization growth, and high technology improvement—would provide the best development model for China to realise socioeconomic sustainable development and a reduction in CO_2_ emissions.

The above findings thus contribute to the literature and suggest meaningful theoretical and policy implications [70–72]. First, from the evidence provided by the results of this study, population scale and urbanization are important factors that increase CO_2_ emissions. Since the Chinese economic reform in 1978, China has entered a period of rapid urbanization. Results suggest that China should continue to carry out the family planning policy, which has aimed to slow the robust growth of the country’s vast population and maintain healthy levels of population urbanization– according to official data, this policy has helped Asia’s biggest economy reduce its population by millions. In addition, China should also continue to promote stable and relative low population urbanization and pay attention to optimizing population structure and quality. More importantly, it is essential for China to make greater efforts to improve low-carbon awareness, advocating for low-carbon consumption and healthy lifestyles and promoting sustainable consumption modes to households. Second, the results of this study clearly indicate that economic level positively influences CO_2_ emissions. Over the past thirty years, China has made great success in economic development, with an annual growth rate of 9.9%. Rapid economic growth can improve living standards, but it can also cause high levels of energy consumption, leading to high CO_2_ emissions. Rapid economic growth, however, retains a high strategic importance for the Chinese government. The government therefore aims to reduce CO_2_ emissions, while still fostering economic growth. The most feasible method to reduce CO_2_ emissions for the Chinese government is not, as such, to sacrifice future economic growth; rather, slowing economic growth relatively constitutes the most effective measure. Third, China pledged to reduce its energy intensity by 20% on average across all provinces by 2010. In the future, China should continue to target cuts in energy intensity. First, emissions reduction indicators, such as energy and emission intensity, should be set relative to physical output rather in terms of economic growth [4]. In addition, it is also important to take various measures such as producing more renewable technologies and low-carbon technologies, boosting recycling and renewable energies, and implementing incentive policies, amongst other measures, in order to strengthen energy conservation and emission mitigation [73].

From the view of methodology, this paper underscores the promising aspects of utilizing panel data models such as panel unit root models, panel cointegration tests, and the Fully Modified OLS in understanding the main determinants of CO_2_ emissions. The results of the panel data models are capable of better revealing the factors hidden in the CO_2_ emissions in Chinese provinces over the period studied. Our empirical analysis of Chinese provinces also demonstrates the appropriateness of setting up different scenario simulations for analyzing the optimal development model for China by addressing the influencing variables’ dynamic evolution changes.

## Supporting Information

S1 Table Raw Data .
(XLSX)

## References

[1] Wang SJ, Fang CL, Ma HT, Wang Y, Qin J. Spatial differences and multi-mechanism of carbon footprint based on GWR model in provincial China. Journal of Geographical Sciences 2014; 24(4): 612–30.

[2] Wang SJ, Fang CL, Guan XL, Ma HT. Urbanization, energy consumption, and CO_2_ emissions in China: a panel data analysis of China’s province. Applied Energy 2014; 136: 738–49.

[3] Wang SJ, Fang CL, Wang Y, Huang YB, Ma HT. Quantifying the relationship between urban development intensity and carbon dioxide emissions using a panel data analysis. Ecological Indicators 2014; 49: 121–31.

[4] Liu Z, Guan DB, Crawford-Brown D, Zhang Q, Liu JG. A low-carbon road map for China. Nature 2013; 500(7461):143–45. doi: 10.1038/500143a 23925225 10.1038/500143a

[5] Guan D, Hubacek K, Weber C, Peters G, Reiner D. The drivers of Chinese CO_2_ emissions from1980 to 2030. Global Environmental Change 2008;18: 626–34

[6] York R, Rosa EA, Dieta T. STIRPAT, IPAT and impact: analytic tools for unpacking the driving forces of environmental impacts. Ecological Economics 2003; 4: 351–65.

[7] Shi A. The impact of population pressure on global carbon dioxide emissions, 1975–l996: evidence from pooled cross-count data. Ecological Economics 2003; 44: 29–42.

[8] Xu ZM, Cheng GD. Impacts of population and affluence on emissions in China. Journal of Glaciology and Geocryology 2005; 27(5):767–73.

[9] Fan Y, Liu LC, Wu G, Tsai HT, Wei YM. Changes in carbon intensity in China: Empirical findings from 1980–2003. Ecological Economics 2007; 62(3): 683–93.

[10] Liu HG, Liu WD. Decomposition of Energy-induced CO_2_ emissions in industry of China. Progress in Geography 2009; 28: 285–92.

[11] Lin SF, Zhao DT, Marinova D. Analysis of the environmental impact of China based on STIRPAT model. Environment Impact Assessment Reviews 2009; 29: 341–7.

[12] Yan H, Guo YG, Lin FC. Analyzing the developing model of Chinese cities under the control of CO_2_ emissions using the STIRPAT model: a case study of Shanghai. Acta Geographica Sinica 2010; 65(8): 983–90.

[13] Shao S, Yang LL, Cao JH. Study on influencing factors of CO_2_ emissions from industrial energy consumption: an empirical analysis based on STIRPAT model and industrial sectors’ dynamic panel data in Shanghai. Journal of Finance Economics 2010; 11: 16–27.

[14] Sun JS, Chen ZR, Li ZJ. A research on influencing factors of low-carbon economy development in China: an analysis based on the extended STIRPAT model. J Audit Econ 2011; 26(4): 85–93.

[15] Li HN, Mu HL, Zhang M, Gui SS. Analysis of regional difference on impact factors of China’s energy-related CO_2_ emissions. Energy 2012; 39: 319–26.

[16] Wang MW, Che Y, Yang K, Wang M, Xiong LJ, Huang YC. A local-scale low-carbon plan based on the STIRPAT model and the scenario method: the case of Minhang District, Shanghai, China. Energy Policy 2011; 39: 6981–90.

[17] Li HN, Mu HL, Zhang M, Li N. Analysis on influence factors of China’s CO_2_ emissions based on path-STIRPAT model. Energy Policy 2011; 39: 6906–11.

[18] Wang ZH, Yin FC, Zhang YX, Zhang X. An empirical research on the influencing factors of regional CO_2_ emissions: Evidence from Beijing city, China. Applied Energy 2012; 100: 277–84.

[19] Ma Z, Liu W, Wang L, Ma PL, Wang YX, Dong DM, Duan HY, Wang XE. Study on energy consumption prediction and energy management in Jilin province based on STIRPAT model. Advanced Materials Research 2013; 281:542–5.

[20] Wang P, Wu WS, Zhu BZ, Wei YM. Examining the impact factors of energy-related CO_2_ emissions using the STIRPAT model in Guangdong Province, China. Applied Energy 2013; 106: 65–71.

[21] Zhao T, Guo X. Analyzing the driving effect of influence factors on CO_2_ emissions using the STIRPAT model in Tianjin of China. Advanced Materials Research 2013; 734: 1896–1900.

[22] Ang BW, Zhang FQ, Choi KH. Factorizing changes in energy and environmental indicators through decomposition. Energy 1998; 23(6): 489–95.

[23] Hoekstra R, Van den Bergh JJCJM. Comparing structural decomposition analysis and index. Energy Economics 2003; 25:39–64.

[24] Wang C, Chen JN, Zou J. Decomposition of energy-related CO_2_ emission in China: 1957–2000. Energy 2005; 30: 73–83.

[25] Wu L, Kaneko S, Matsuoka S. Driving forces behind the stagnancy of China’s energy-related CO_2_ emissions from 1996 to 1999: the relative importance of structural change, intensity change and scale change. Energy Policy 2005; 33: 319–35.

[26] Ma C, Stern DI. Biomass and China’s carbon emissions: A missing piece of carbon decomposition. Energy Policy 2008; 36: 2517–26.

[27] Elif A, Gül IT, Serap TA. CO_2_ emissions of Turkish manufacturing industry: a decomposition analysis. Applied Energy 2011; 88: 2273–8.

[28] Song JK. Factor decomposition of carbon emissions from energy consumption of Shandong Province based on LMDI. Resource Science 2012; 34(1): 35–41.

[29] Zhang L, Lei J, Zhou X, Zhang XL, Dong W, Yang Y. Changes in carbon dioxide emissions and LMDI-based impact factor decomposition: the Xinjiang Uygur autonomous region as a case. Journal of Arid Land 2014; 6(2): 145–55.

[30] Brizga J, Feng KS, Hubacek K. Drivers of greenhouse gas emissions in the Baltic States: A structural decomposition analysis. Ecological Economics 2014; 98: 22–8.

[31] Gui SS, Mu HL, Li N. Analysis of impact factors on China's CO_2_ emissions from the view of supply chain paths. Energy 2014; 74: 405–16

[32] Xu SC, He ZX, Long RY. Factors that influence carbon emissions due to energy consumption in China: Decomposition analysis using LMDI. Applied Energy 2014; 127: 182–193.

[33] Du LM, Wei C, Cai SH. Economic development and carbon dioxide emissions in China: Provincial panel data analysis. China Economic Reviews 2012; 23(2): 371–84.

[34] Al-mulali U. Factors affecting CO_2_ emission in the Middle East: A panel data analysis. Energy 2012; 4(1):564–9.

[35] Dinda S, Coondoo D. Income and emission: A panel data-based cointegration analysis. Ecological Economics 2006; 57(2):167–81

[36] Narayan PK, Narayan S. Carbon dioxide emissions and economic growth: Panel data evidence from developing countries. Energy Policy 2010; 38(1): 661–6.

[37] Wang SS, Zhou DQ, Zhou P, Wang QW. CO_2_ emissions, energy consumption and economic growth in China: A panel data analysis. Energy Policy 2011; 39(9): 4870–5.

[38] Ozkan F, Ozkan O. Panel data analysis for the CO_2_ emissions, the industrial production and the energy sector of the OECD countries. Energy Education Science and Technology Part A- Energy Science and Research 2012; 29(2):1233–44.

[39] Zhu HM, You WH, Zeng ZF. Urbanization and CO_2_ emissions: A semi-parametric panel data analysis. Economic Letters 2012; 117(3): 848–50

[40] Ozcan B. The nexus between carbon emissions, energy consumption and economic growth in Middle East countries: A panel data analysis. Energy Policy 2013; 62:1138–47.

[41] Liang QM, Fan Y, Wei YM. Multi-regional input–output model for regional energy requirements and CO_2_ emissions in China. Energy Policy 2007; 35(3): 1685–1700.

[42] Zhang ZX. Why did the energy intensity fall in China’s industrial sector in the 1990s? The relative importance of structural change and intensity change. Energy Economics 2003; 25: 625–38.

[43] Xu GQ, Liu ZY, Jiang ZH. Decomposition model and empirical study of carbon emissions for China, 1995–2005. China Population Resource and Environment 2006; 16(6):158–61.

[44] Zha DL, Zhou DQ. The inequality about provincial energy efficiency and its related CO_2_ emission: decomposition based on kaya. System Engineer 2007; 25(11): 65–71.

[45] Liu YB. Exploring the relationship between urbanization and energy consumption in China using ARDL (autoregressive distributed lag) and FDM (factor decomposition model). Energy 2009; 34:1846–54.

[46] Shi LY, Zhang HW. Factor analysis of CO_2_ emission changes in China. Energy Procedia 2011; 5: 79–84.

[47] Hawkes AD. Long-run marginal CO_2_ emissions factors in national electricity systems. Applied Energy 2014; 125: 197–205.

[48] Zhang M, Wang WW. Decouple indicators on the CO_2_ emission-economic growth linkage: The Jiangsu Province case. Ecological Indicators 2013; 32: 239–44.

[49] Liu YB, Xie YC. Asymmetric adjustment of the dynamic relationship between energy intensity and urbanization in China. Energy Economics 2013; 36: 43–54.

[50] IPCC guidelines for national greenhouse gas inventories Intergovernmental panel on climate change. IPCC, London, 2006.

[51] National Coordination Committee Office on Climate Change and Energy Research Institute under the National Development and Reform Commission. National greenhouse gas inventory of the People's Republic of China. Beijing: Chinese Environmental Science Press, 2007.

[52] Levin A, Lin C, Chu C. Unit root tests in panel data: asymptotic and finite-sample properties. Journal of Econometrics 2002; 108(1):1–24.

[53] Breitung J. The local power of some unit root tests for panel data. Advances Econometrics 2000; 15: 161–77.

[54] Im KS, Pesaran MH, Shin Y. Testing for unit roots in heterogeneous panels. Journal of Econometrics 2003; 115(1): 53–74.

[55] Pedroni P. Panel cointegration: asymptotic and finite sample properties of pooled time series tests, with an application to the PPP hypothesis: new results Working paper, Indiana University, 1999.

[56] Li F, Dong SC, Li X, Liang QX, Yang WZ. Energy consumption-economic growth relationship and carbon dioxide emissions in China. Energy Policy 2011; 39(2):568–74.

[57] Phillips PCB, Perron P. Testing for a unit root in time series regression. Biometrika 1988; 75(2): 335–46.

[58] Pedroni P. Fully modified OLS for heterogeneous cointegrated panels. Advanced Econometrics 2000; 15: 93–130.

[59] International Energy Agency (IEA) (2007). World energy model-methodology and assumptions. Available at: http://www.worldenergyoutlook.org/docs/weo2007/WEM_Methodology_07.pdf

[60] Soytas U, Sari R, Ewing BT. Energy consumption, income, and carbon emissions in the United States. Ecological Economics 2006; 62(3: 482–9

[61] Hamit-Haggar M. Greenhouse gas emissions, energy consumption and economic growth: a panel cointegration analysis from Canadian industrial sector perspective. Energy Economics 2012; 34(1): 358–64.

[62] Niu SW, Ding YX, Niu YZ, Li YX, Luo GH. Economic growth, energy conservation and emissions reduction: a comparative analysis based on panel data for 8 Asian-Pacific countries. Energy Policy 2011; 39(4): 2121–31.

[63] Pao HT, Tsai CM. CO_2_ emissions, energy consumption and economic growth in BRIC countries. Energy Policy 2010; 38(12): 7850–60.

[64] Kum H, Ocal O, Aslan A. The relationship among natural gas energy consumption, capital and economic growth: bootstrap-corrected causality tests from G-7countries. Renewable and Sustainable Energy Reviews 2012; 16(5): 2361–5.

[65] Chen WY, Gao PF, He JK. Impact of carbon mitigation on China's energy system using China MARKAL-MACRO model. Journal of Tsinghua University (Sci & Tech) 2004; 44(3): 342–6.

[66] International Energy Agency (IEA). World energy outlook. Paris: IEA, 2008.

[67] Cai WJ, Wang C, Chen JJ, Wang K, Zhang Y, Lu XD. Comparison of CO_2_ emissions scenarios and mitigation opportunities in China's five sectors in 2020. Energy Policy 2008; 36(3):1181–94.

[68] Energy Information Administration (EIA), 2009. International energy outlook. Available at: http://www.eia.doe.gov/oiaf/ieo/.

[69] Energy Research Institute under National Development and Reform Commission (ERI) (2009). China's low carbon development pathways by 2050: Scenario analysis of energy demand and carbon emissions. Beijing: Science Press.

[70] Pao H, Tsai C. Modeling and forecasting the CO_2_ emissions, energy consumption, and economic growth in Brazil. Energy 2011; 36: 2450–8.

[71] Pao H, Yu H, Yang Y. Modeling the CO_2_ emissions, energy use, and economic growth in Russia. Energy 2011; 36:1–7.

[72] Wang SJ, Ma HT, Zhao YB. Exploring the relationship between urbanization and the eco-environment—a case study of Beijing–Tianjin–Hebei region. Ecological Indicators 2014; 45: 171–83.

[73] Zhao YB, Wang SJ. The relationship between urbanization, economic growth and energy consumption in China: an econometric perspective analysis. Sustainability, 2015; 7, 5609–5627.

